# The Role of Acquired Immunity in the Spread of Human Papillomavirus (HPV): Explorations with a Microsimulation Model

**DOI:** 10.1371/journal.pone.0116618

**Published:** 2015-02-02

**Authors:** Suzette M. Matthijsse, Joost van Rosmalen, Jan A. C. Hontelez, Roel Bakker, Inge M. C. M. de Kok, Marjolein van Ballegooijen, Sake J. de Vlas

**Affiliations:** 1 Department of Public Health, Erasmus MC, University Medical Center Rotterdam, Rotterdam, the Netherlands; 2 Department of Biostatistics, Erasmus MC, University Medical Center Rotterdam, Rotterdam, the Netherlands; Georgetown University, UNITED STATES

## Abstract

**Background:**

Knowledge of the natural history of human papillomavirus (HPV), in particular the role of immunity, is crucial in estimating the (cost-) effectiveness of HPV vaccination and cervical cancer screening strategies, because naturally acquired immunity after clearing an infection may already protect part of the risk population against new HPV infections.

**Methods:**

We used STDSIM, an established stochastic microsimulation model, quantified to the Netherlands. We explored different assumptions regarding the natural history of HPV-16 and HPV-18, and estimated the transmission probabilities and durations of acquired immunity necessary to reproduce age-specific prevalence.

**Results:**

A model without acquired immunity cannot reproduce the age-specific patterns of HPV. Also, it is necessary to assume a high degree of individual variation in the duration of infection and acquired immunity. According to the model estimates, on average 20% of women are immune for HPV-16 and 15% for HPV-18. After an HPV-16 infection, 50% are immune for less than 1 year, whereas 20% exceed 30 years. For HPV-18, up to 12% of the individuals are immune for less than 1 year, and about 50% over 30 years. Almost half of all women will never acquire HPV-16 or HPV-18.

**Conclusions:**

Acquired immunity likely plays a major role in HPV epidemiology, but its duration shows substantial variation. Combined with the lifetime risk, this explains to a large extent why many women will never develop cervical cancer.

## Introduction

Infection with human papillomavirus (HPV) is a necessary cause for developing cervical cancer [1], the fourth most common cancer among women worldwide [2]. HPV is one of the most prevalent sexually transmitted infections (STI) [3,4], and over 118 different types have been identified [5]. At least 14 of these are considered high-risk types for cervical cancer due to their oncogenic nature after progressing for a longer period of time [6]. However, most HPV infections clear naturally. In the Netherlands, HPV prevalence in the general population (among women with normal cytology) was estimated to be 3.9% in 2010, with a peak in younger women [7].

The bivalent vaccine Cervarix offers protection against HPV-16 and HPV-18, which account for 70–76% of the cervical cancers [8]. Cervarix was introduced in the Netherlands in 2009 for 12-year old girls with a catch-up campaign for girls aged 13–16 years, with a modest vaccine uptake of only 50% [9]. The vaccine was implemented in the national immunization program in 2010. A crucial factor in estimating the effect of vaccination is naturally acquired immunity, yet little is known about its degree and duration [10]. Understanding the transmission and immunity mechanisms of HPV is essential to adequately predict the (cost-)effectiveness of HPV vaccinations and cervical cancer screening strategies.

Several mathematical models have been developed to study the spread of HPV and immunity processes responsible for clearing an HPV infection [11–17]. Some of these models are deterministic [13,14,17], and may not properly account for the complexity of transmission through sexual networks [18]. Also, only HPV prevalence data of limited age ranges are used [16], or the transmission probability was estimated per sexual partnership instead of per sexual contact [11,13,14]. An exception is the study by Burchell *et al*. (2006), who estimated the median transmission probability at 40% per sex act, but ranging from a lower limit of 5% to an upper limit of 100% [15]. This wide range indicates that further research on the transmission probability per sexual contact is necessary, ideally by using more detailed data.

We explored the transmission dynamics of HPV-16 and HPV-18, especially regarding the process of acquired immunity, by reproducing the age patterns of type-specific HPV prevalence in the Netherlands [19,20], using the established stochastic microsimulation model STDSIM. This model has been used extensively for heterosexual transmission and control of HIV and other STIs in African settings [21–23]. In the current study, we quantified the model to a Western setting for the first time. The transmission of chlamydia ([24], personal communication), of which more is known regarding its natural history, has been included in the model to validate our model fit of sexual behavior.

## Methods

### STDSIM and its quantification to the Netherlands

STDSIM simulates the life course of individuals in a dynamic network of heterosexual contacts, in which STIs, such as HPV, can spread. Each individual has its own characteristics that are either constant (e.g., date of birth and sex) or subject to change (e.g., number of sexual partners, infection status). Events are determined by probability distributions, and can lead to new events (e.g., a birth leads to a future event of becoming sexually active) or a cancellation of future events (e.g., a death cancels all scheduled events concerning sexual activity or STI transmission for this person and to or from his/her partner). More information on the model structure can be found in the S1A Supporting Information.

We first used generally available information to quantify our model regarding demography and sexual behavior. Age- and sex-specific life expectancy and migration data [25] and age-specific fertility rates [25,26] were used to quantify STDSIM such that it represents the Dutch demography. To model the sexual network structure, we used data from three national sexual health surveys [27–29]. We then simulated the transmission of chlamydia. Chlamydia is a short-lasting STI that is common among heterosexuals in the Netherlands, and was included to validate the level of risk behavior in the model. A detailed description of the model quantification for the Dutch setting and the transmission and natural history of chlamydia can be found in S1B-S1D Supporting Information.

### Transmission and natural history of HPV

We simulated the transmission of HPV-16 and HPV-18. We assumed equal transmission probabilities per sexual contact for male-to-female and female-to-male transmission [13,15,17]. The transmission of both HPV types is independent given individual sexual risk behavior, and HPV transmission parameters were calibrated to make the model fit the observed age- and type-specific HPV prevalences.

Two important aspects distinguish the transmission of HPV from other STIs in our model. First, transmission can also occur through genital skin [30], and previous research shows no association between condom use and HPV acquisition risk [31]. Therefore, we assumed that condoms do not have a protective effect against HPV, though they do protect against chlamydia. Second, we assumed that women who have had a hysterectomy cannot acquire an HPV infection, similar to other modeling studies [13,14]. We used age-specific data to model the fraction of women in the population who have had a hysterectomy [32]. In the model, women clear an existing HPV infection immediately after a hysterectomy, and are considered immune for future HPV infections.

We further assumed that the durations of infection and acquired immunity follow a Weibull distribution. The Weibull distribution is a continuous probability distribution, defined by a shape and scale parameter. If the shape parameter is set to 1, the distribution reduces to the exponential distribution. The shape parameters were varied with pre-set values of 0.25, 0.50, 1, 2 and 4. This allowed us to explore whether large (small shape parameter) or little (high shape parameter) individual variation in the average duration of infection and acquired immunity would be consistent with the available data.

The mean duration of an HPV infection in women was based on the studies of Goodman *et al*. [33] and Trottier *et al*. [34], which measured type-specific time to clearance of an incident infection in women with normal cytology within a large age range. Together, these studies resulted in a total sample size of 966 women aged 18–85 years. We first transformed the median durations reported by Goodman *et al*. [33] into mean durations, depending on the shape parameter of the Weibull distribution assumed. A weighted average of these results and mean durations as reported in Trottier *et al*.[34] was then used as the mean durations for each HPV-type in our model (S3 Table). To obtain mean infection durations in men, we again transformed median durations based on the *HPV in Men* (HIM) Study of Giuliano *et al*. [35], which included 1159 men aged 18–70 years.

In the base case analysis, we assumed that everyone acquires full immunity for some (Weibull distributed) time immediately after clearing an infection. During this period, an individual cannot acquire a new HPV infection, irrespective of whether this is due to the development of antibodies or other components of the adapted immune system. We varied the immunity duration from no immunity to effectively lifelong, and the transmission probability from 0% to 100%, to find parameter values that made the model most accurately reproduce the observed HPV prevalence trends with age (see section “Optimizing HPV parameters”). In addition, we assumed an alternative mechanism for acquired immunity based on the history of HPV infections, for which the methods and results are documented in S1E Supporting Information.

We used data on age-specific prevalence of high-risk HPV infections among women in the Netherlands from the POBASCAM study [20]. The POBASCAM study is a population-based randomized controlled trial (recruitment of women from 1999–2002) on the implementation of high-risk HPV testing in cervical screening, including 21,950 Dutch women between the ages of 29–61 years [20]. We added data from a cross-sectional study (2007) among women aged 18–29 years representative for the general population in the Netherlands, to include HPV prevalence data of young women outside the screening age [19]. Both studies only measured the overall high-risk HPV prevalence. Based on Coupé *et al*., who also used POBASCAM data [36], we calculated the age-specific fractions of each HPV type within the overall prevalence, and applied these fractions to the above mentioned data to obtain the type-specific HPV prevalence (S4 Table). We assumed a 94% HPV test sensitivity [37,38].

### Optimizing HPV parameters

We performed a grid search to determine combinations of transmission probabilities and mean duration of acquired immunity (Weibull distributed) for each HPV-type, and the 5 pre-set shape parameters of the Weibull distributions of the durations of infection and acquired immunity that result in the best fit to the type- and age-specific HPV prevalence. For each parameter combination, we ran the model 100 times. The best fit was based on the binomial log-likelihood of the observed prevalence data and model estimates summed over the age categories. The log-likelihood ratio is the difference between log-likelihoods of two different models [39]. To compare models, we performed likelihood ratio tests using the log-likelihood ratio times-2, which is approximately chi-squared distributed.

For the combinations of Weibull shapes with the best fit, we used a regression model of the transmission probability and acquired immunity duration as a metamodel [39,40]. First, we fitted the log-likelihoods of the grid to a second order polynomial regression model, as has been done by Fischer *et al*. [39]. We then determined the optimal parameter combination of the transmission probability and acquired immunity duration, as well as 95% confidence intervals (CIs). We ran the model with this optimal parameter combination 1000 times, to precisely determine the predicted average age- and type-specific HPV prevalence. Finally, we performed a chi-squared test summed over the age categories to determine the goodness-of-fit of our model compared to the observed HPV prevalence.

Finally, we used the optimal model to estimate the age-specific proportion of women who are immune for HPV-16 and HPV-18, respectively. We accounted for uncertainty in the type-specific transmission probability and the duration of acquired immunity by re-running the model with 200 alternative parameter combinations [23]. We randomly sampled combinations from the 95% CIs of the estimated transmission probabilities and immunity durations, and accepted them if the resulting age-specific HPV prevalence did not differ significantly from the optimal model predictions. Uncertainty ranges for the proportion of women with acquired immunity were subsequently obtained by taking the 5^th^ lowest value as lower bound and 5^th^ highest value as upper bound for each age group.

## Results

**Figure 1:**
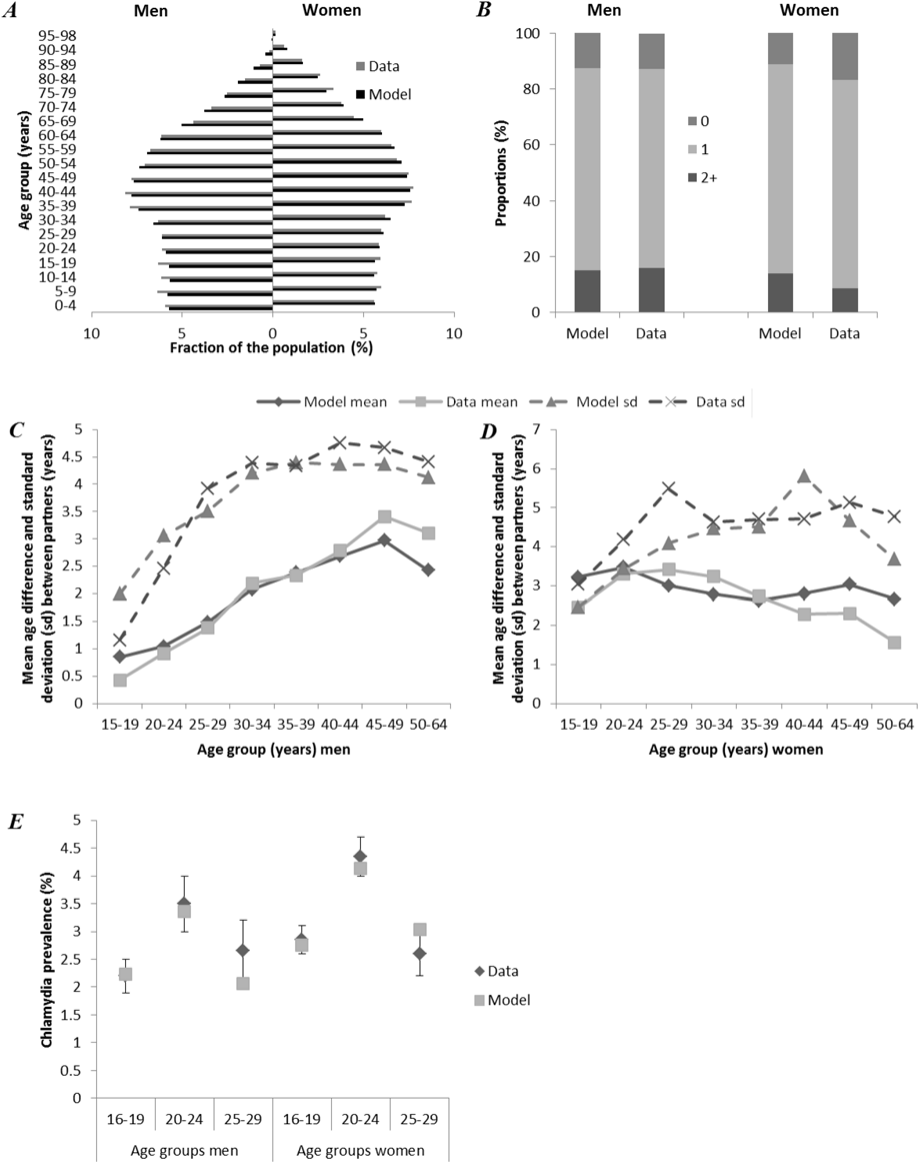
Comparison of the model predictions with data of population composition, sexual behavior and chlamydia prevalence. Modeled population composition per age group, compared to data of Statistics Netherlands [25] ***(A)***. Modeled number of partners in the last 6 months in men and women aged 20–64 years, compared to data of 19–64 years old in Rutgers WPF [27,28] ***(B)***. Mean and standard deviations (sd) of age differences in relationships, reported by male respondents ***(C)*** and female respondents ***(D)***. Modeled chlamydia prevalence of 15–29 years old compared to the data of Van den Broek *et al*. (2012) [24] of 16–29 years old ***(E)***.

Our model is able to accurately simulate the Dutch population (Fig. 1A), number of recent partners for men (Fig. 1B, left bars) and age difference in relationships (Fig. 1C and 1D). The model does show a larger proportion of women with 2 or more recent partners than reported in the surveys (Fig. 1B, right bars). However, especially for women, it is well-known that social desirability bias in self-reported sexual behavior often results in underreporting of the number of current or recent partners to make a more favorable appearance [41,42]. The modeled prevalence of chlamydia for both men and women is close to the observed prevalence levels given by Van den Broek *et al*. ([24], personal communication; Fig. 1E).

**Figure 2:**
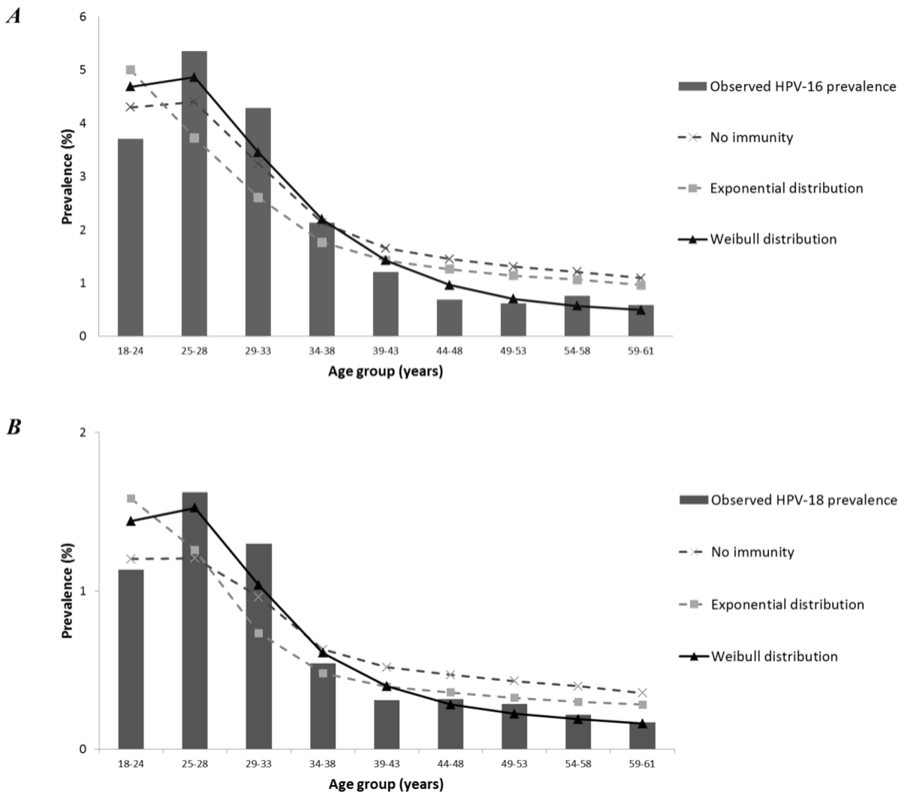
Comparison of the observed and estimated age-specific HPV prevalence. The estimated prevalence is given by the best fitting models of the different scenarios. These scenarios include no acquired immunity after clearing an infection (exponential distribution for the duration of infection); exponentially distributed durations; Weibull distributed durations. ***(A)*** shows the results for HPV-16; ***(B)*** for HPV-18.

Fig. 2 shows the best estimated HPV-16 and HPV-18 prevalence when assuming no immunity, exponential distributions, and Weibull distributions. A model without acquired immunity clearly cannot reproduce the age-specific patterns in the prevalence of HPV-16 and HPV-18 (Fig. 2). The prevalence is underestimated in women aged 25–33 years, while overestimated in older age groups. For both HPV-types, it is also not sufficient to assume exponential distributions for the duration of infection and acquired immunity. While the best fitting model for HPV-16 still deviates from the data for women aged 18–33 years (χ^2^ (5) = 15.26, *p* =.009; S1F Supporting Information, S5 Table), it provides a significantly better fit than the model without immunity (χ^2^ (4) = 43.88, *p*<.001), and the model with exponential distributions (χ^2^ (4) = 53.24, *p* <.001). This best fitting model had Weibull shape parameters of 0.50 and 0.25 for the duration of infection and immunity, respectively, corresponding with half of the men and over 70% of women clearing their infection within 1 year (S3 Table). In this model, almost 50% of the individuals are no longer immune 1 year after clearing the infection (S6 Table). Still, about 20% have an estimated duration of acquired immunity that lasts longer than 30 years. The corresponding estimated transmission probability is 6.9% per sexual contact (95% CI: 5.4; 8.6).

For HPV-18, we found similar individual variation in the infection duration compared to HPV-16. The corresponding Weibull shape of 0.50 suggests that 62% of men and 69% of women clear their infection within 1 year (S3 Table). For almost 15% of men and over 9% of women, infection lasts longer than 4 years. The model with immunity fits the data significantly better than the model without immunity, in which the prevalence is underestimated in women aged 25–33 years and overestimated in women aged 34+ years (Fig. 2B, S5 Table). The model with Weibull distributions fits the data better than with exponential distributions, which shows similar deviations as the model without immunity (Fig. 2B, S5 Table). Fits using Weibull shapes of 0.50, 1, 2, and 4 are comparable, indicating that the shape parameter of the duration of immunity is less important for HPV-18 compared to HPV-16. These shape parameters correspond with 42–50% of the individuals having an estimated duration of acquired immunity longer than 30 years, while only 0–12% is immune for less than 1 year after clearing the infection (S6 Table). Corresponding estimated transmission probabilities for the model range from 6.7% (95% CI: 5.4; 8.3) to 9.0% (95% CI: 5.7; 13.0) per sexual contact. This model shows no significant deviations from the observed HPV-18 prevalence (S5 Table).

**Figure 3:**
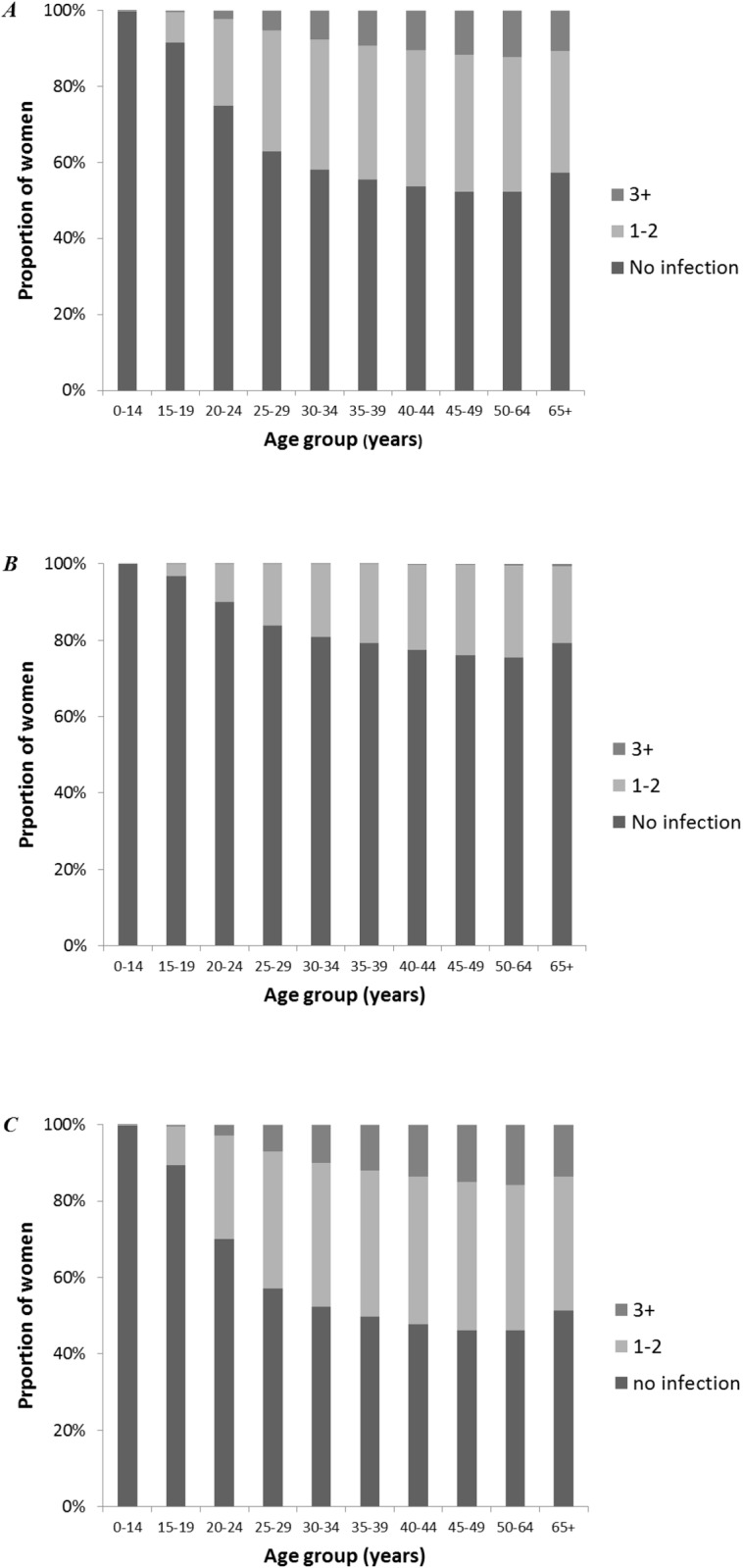
Predicted cumulative number of HPV-16 *(A)*, HPV-18 *(B)*, and HPV-16/-18 *(C)* infections in women. The proportion of women with no lifetime infections slightly increased in women aged 65+ compared to women aged 50–64 years. This results from a cohort effect due to a combination of historical data on fertility rates and timing of an increase in migration (1965), and will only have a minimal effect on our estimates.

**Figure 4:**
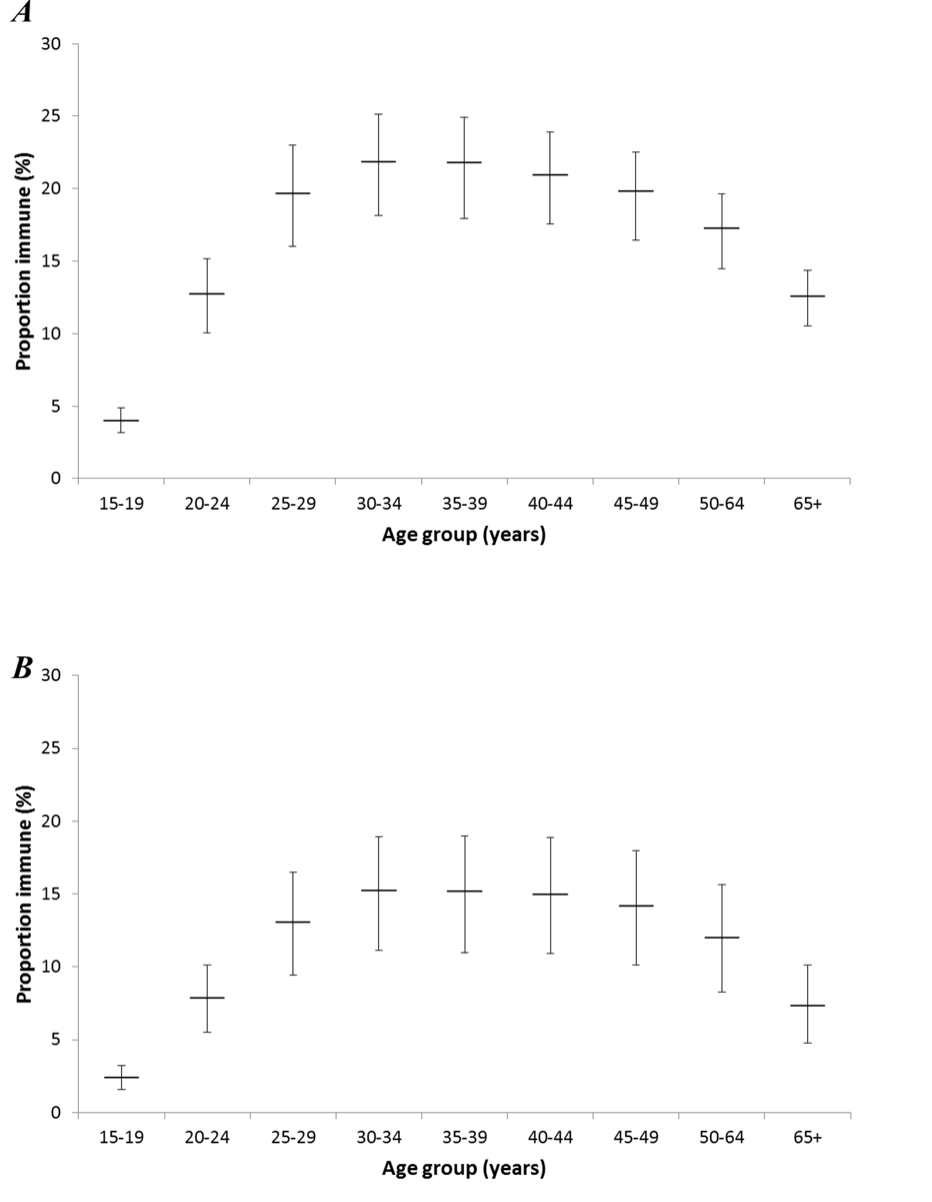
The distribution of acquired immunity in the best fitting model for HPV-16 *(A)* and HPV-18 *(B)*. Bars reflect uncertainty ranges (see Methods).

The best fitting model for HPV-16 and HPV-18 suggests that approximately half of the women in the model will have an HPV-16 infection in their lifetime (Fig. 3A), and 25% an HPV-18 infection (Fig. 3B). About 46% of women will never acquire either an HPV-16 or HPV-18 infection in our model (Fig. 3C). The proportion of women who are immune to HPV-16 is higher than HPV-18, peaking at 22% of women aged 30–39 years for HPV-16 (Fig. 4A) and 15% of women aged 30–44 years for HPV-18 (Fig. 4B).

## Discussion

In this study we quantified STDSIM to a high-income country for the first time. The model was able to accurately replicate the Dutch population and sexual network. Our estimates indicate that including a mechanism of acquired immunity is essential to adequately explain the age patterns of HPV prevalences in the Netherlands. Also, our model suggests that there is large individual variation in the duration of infection and acquired immunity. This is especially the case for HPV-16, for which, in our model, 50% of the individuals are immune only for less than a year after clearing an infection, yet 20% for longer than 30 years. For HPV-18, a higher proportion will develop long lasting acquired immunity.

Our finding that large individual variation exists in naturally acquired immunity is consistent with Mikolajczyk *et al*. [43], who concluded that the observed distribution of antibody titers reflects individual differences in immune response. We found that, especially for HPV-16, most women only become immune for a relatively short period of time (less than 1 year) after clearing an infection. This concurs with earlier findings of an association between re-infections and sexual activity [44], likely due to rapidly waning immunity after clearing an infection in some women. In addition, studies found that only 40–60% of previously infected individuals have detectable antibodies shortly after an infection [45,46], further suggesting that many women were immune for a short period of time or not at all. Surprisingly, our results suggest that acquired immunity lasts longer than 30 years for about half of the people after clearing an infection of HPV-18, whereas the proportion of people with acquired immunity less than 1 year is minimal, ranging from 0.07% to 12% in the best fitting model. Baussano *et al*. [47] also found in his model that a larger proportion of individuals developed lifelong immunity for HPV-18 compared to HPV-16. It could be that, for those developing naturally occurring antibodies, the amount of protection may be different between HPV-16 and HPV-18 [48]. Possible reasons behind these contrasting findings should follow from clinical research.

We also found that some degree of individual variation must be assumed in the duration of infection. This variation in infection duration could to some extent be attributable to latent undetectable infections and might be difficult to measure in observational studies. While some infections might appear as two separate infections in observational studies, they could be an ongoing long-term infection that might have been latent during follow-up visits. On the other hand, very short infections might not be detected since they could fall between follow-up visits [49].

Our model for HPV-18 has a good model fit and does not deviate significantly from the observed prevalence data. For HPV-16, the model shows a slightly worse fit to the data. In particular, it overestimates the HPV-16 prevalence for women aged 18–24 years, and shows a small underestimation for women aged 25–33 years. This may be due to dynamics in natural history specific to HPV-16 that are difficult to capture in models. However, the differences are relatively small and are unlikely to influence our results.

Our study has some limitations. First, we ignored effects as a result of cervical cancer and cervical cancer screening on HPV transmission. Infections only clear naturally in our model and not through the removal of high-grade lesions. However, the majority of infections does not end by treatment of cervical neoplasia. Hence, the underestimation of the transmission probability per sexual contact is likely minimal. Second, the Weibull shapes in our model for the duration of infection and acquired immunity are only fitted using pre-set parameter values. Limited data did not allow a more precise assessment of shapes. Thus, individual variation in reality could somewhat differ from the best fitting model parameters chosen in our study. Third, we assumed that the durations of infection and immunity are randomly drawn. It could be that these durations depend on individual characteristics, such as age, history of HPV infections, or individual factors related to the immune system. Fourth, we did not incorporate cross-protection of immunity between HPV-16 and HPV-18 in our model. Clinical trials have shown that the HPV-specific vaccines protect to some extent against other HPV-types as well [50], indicating that cross-protection may also exist in naturally acquired immunity. However, the extent of cross protection of acquired immunity against HPV-16 and HPV-18 is currently unknown, and we have therefore not incorporated it in our model. A particular strength of our study is that we did not rely on HPV prevalence data based on opportunistic cervical screening for younger women (outside of the age range of the Dutch cervical cancer screening program). A representative sample was used instead, leading to more reliable age-specific patterns which are crucial in understanding the natural history of HPV.

Our model suggests that the lifetime risk of acquiring an HPV-16 or HPV-18 infection for women is about 50%. This is nearly as high as the risk of only HPV-16, indicating that many women with an HPV-18 infection will also acquire an HPV-16 infection in their lifetime. In our model, acquired immunity for HPV-16 and HPV-18 infections peaks at 22% and 15% of women respectively, and especially those with long-term acquired immunity will benefit less from vaccination as they are already protected naturally. In addition, women who develop immunity are the women with the highest risk, given their history of an HPV infection. Therefore, even though HPV vaccination will certainly decrease individual risks of cervical cancer, many women would not really need it since they will never acquire an HPV-16 or HPV-18 infection during their lifetime, and others are already protected because of naturally acquired immunity after clearing an infection. Clearly, vaccines will be most effective when targeted at young women, at an age before starting sexual relationships and being exposed to HPV for the first time. As the indirect effects of vaccination depend on the sexual network, acquired immunity, vaccine coverage, and target groups, a model is essential to further study the (cost-)effectiveness and optimal delivery strategies of HPV vaccination in preventing cervical cancer.

We also explored an alternative mechanism for acquired immunity after clearing an HPV-16 or HPV-18 infection (S1E Supporting Information). In this mechanism, women are not fully immune for variable duration, yet their susceptibility to re-infection reduces cumulatively after each infection. For both HPV-types, we found similar individual variation in infection duration and transmission probabilities compared to the base case mechanism. It was again necessary to assume acquired immunity after clearing an infection. Also under this mechanism, about half of the women will never acquire HPV-16 or -18. The alternative mechanism did show a worse fit to the observed prevalence data.

In conclusion, we show that, using a mathematical model, acquired immunity likely plays a major role in HPV epidemiology. While our model suggests that most people are only immune for a short period of time after clearing an HPV-16 infection, acquired immunity is long-term for most people after clearing an HPV-18 infection. The proportion of women being immune for HPV-16 and HPV-18 and lifetime risk for an HPV-16 and HPV-18 infection already explains to a large extent why most women will never develop cervical cancer.

## Supporting Information

S1 Supporting Information The role of acquired immunity in the spread of human papillomavirus (HPV): explorations with a microsimulation model. (DOCX)

S1 Fig Comparison of the observed and estimated age-specific HPV prevalence. The estimated prevalence is given by the best fittings model when assuming no immunity (exponential distribution for the duration of infection) or different scenarios when assuming cumulatively decreasing susceptibility to re-infection after each infection (58% for HPV-16 and 80% for HPV-18). These scenarios include an exponentially distributed duration of infection and a Weibull distributed duration of infection (Weibull shape 0.50). ***(A)*** shows the results for HPV-16; ***(B)*** for HPV-18.
(TIF)

S2 Fig Predicted cumulative number of HPV-16 *(A)*, HPV-18 *(B)*, and HPV-16/-18 infections in women. The proportion of women with no lifetime infections slightly increased in women aged 65+ compared to women aged 50–64 years. This results from a cohort effect due to a combination of historical data on fertility rates and timing of an increase in migration (1965), and will only have a minimal effect on our estimates.
(TIF)

S1 Table Sexual behavior parameters adjusted from previous STDSIM applications in order to reproduce to Dutch sexual network. (DOCX)

S2 Table Age preference matrix for men and women, adjusted to reproduce the observed age differences in relationships. (DOCX)

S3 Table The duration of infection when assuming different values for the shape parameter of the Weibull distribution of HPV-16 and HPV-18 infections. The distribution is a weighted average based on the studies of Trottier *et al*. and Goodman *et al*. Bold numbers indicate the Weibull shape and corresponding duration of infection for the best fitting models.
(DOCX)

S4 Table Observed high-risk HPV (hrHPV) prevalence with the type-specific fractions and the corresponding type-specific prevalence per age group. The observed high-risk HPV prevalence is based on the studies of Lenselink *et al*. and Bulkmans *et al*. By applying the fractions observed in Coupé *et al*., we obtained the type-specific HPV-16 and HPV-18 prevalence.
(DOCX)

S5 Table Parameter values and goodness-of-fit for the best fitting HPV-16 and HPV-18 models of the different scenarios and both acquired immunity mechanisms. The scenarios include no acquired immunity; exponentially distributed durations (Weibull shape = 1); and Weibull distributed durations.
(DOCX)

S6 Table Parameter values and the distribution of immunity durations for the best fitting HPV-16 and HPV-18 models under the base case and alternative immunity mechanism. Scenarios include no acquired immunity; exponentially distributed duration (Weibull shape = 1); and Weibull distributed duration.
(DOCX)
