# Supplementary material for: The Role of Acquired Immunity in the Spread of Human Papillomavirus (HPV): Explorations with a Microsimulation Model
Source: PLoS One. 2015 Feb 2;10(2):e0116618. doi: 10.1371/journal.pone.0116618 (PMC4314063; doi:10.1371/journal.pone.0116618)
Supplement: S1 Supporting Information — (DOCX) [file pone.0116618.s001.docx]

The role of acquired immunity in the spread of human papillomavirus (HPV): explorations with a microsimulation model

Supporting Information S1

Suzette M. Matthijsse^1*^, Joost van Rosmalen^2^, Jan A.C. Hontelez^1^, Roel Bakker^1^, Inge M.C.M de Kok^1^, Marjolein van Ballegooijen^1^, Sake J. de Vlas^1^

^1^Department of Public Health, Erasmus MC, University Medical Center Rotterdam, Rotterdam, the Netherlands

^2^Department of Biostatistics, Erasmus MC, University Medical Center Rotterdam, Rotterdam, the Netherlands

*Corresponding author: P.O. Box 2040

3000 CA Rotterdam

The Netherlands

E-mail: [s.matthijsse@erasmusmc.nl](mailto:s.matthijsse@erasmusmc.nl)

Telephone number: +31 10 704 31 12

Fax number: +31 10 703 84 75.

S1A: General model structure

S1B: Demography

S1C: Sexual behavior

S1D: Chlamydia

S1E: Alternative acquired immunity mechanism

S1F: Parameter values and goodness-of-fit of the best fitting models for HPV-16 and HPV-18

**S1A: General model structure**

STDSIM has four main modules: (1) demography, (2) sexual behavior, (3) STI transmission and natural history, and (4) interventions [[1-4](#_ENREF_1)]. The demography module contains processes that determine the demographic structure of the simulated population, such as fertility, mortality, and migration. The sexual behavior module includes the processes of starting and ending relationships, frequency of sexual contacts, and age mixing patterns. The module of STI transmission and natural history defines the duration of disease stages, STI symptoms, transmission probabilities per sexual contact and possible immunity processes. Finally, the interventions module describes the timing, effectiveness and further consequences of treatments, as well as condom use.

Thus far, STDSIM has only been used for sub-Sahara African settings [[1-3](#_ENREF_1)]. We quantified the model for the first time to a Western situation, by adjusting parameters of demography, sexual behavior, and chlamydia transmission, and by including HPV transmission, HPV natural history and hysterectomies based on observational data for the Netherlands. Details regarding these adjustments, as well as an alternative mechanism for acquired immunity against HPV, goodness-of-fit procedures, and parameter values for the best fitting models of HPV-16 and HPV-18 are given below.

**S1B: Demography**

We used data from Statistics Netherlands to reproduce an average Dutch population [[5](#_ENREF_5)]. Model runs start in 1911 with almost 10,000 men and over 10,000 women. New individuals enter the model through birth and immigration, while deaths and emigration remove individuals from the population. New births are randomly assigned to sexually active women in the age range 15-49 years. We used age-specific fertility rates to assign births proportionally to observed fertility patterns across different age-groups [[5](#_ENREF_5),[6](#_ENREF_6)]. After birth, each individual is assigned a date of death, which is drawn from a pre-defined life-table. We used life expectancy data of the Dutch population in 2008 to construct an age- and sex-specific life table representative for the general population in the Netherlands [[5](#_ENREF_5)]. Finally, we used the average of the age- and gender-specific migration rates of the Dutch population from 2000-2008 to simulate immigration and emigration [[5](#_ENREF_5)]. The simulated population consists of about 80,000 men and women in 2008. The simulated population was compared to data of the population composition of the Netherlands in 2008 (Figure 1A)[[5](#_ENREF_5)].

**S1C: Sexual behavior**

We adjusted model parameters for sexual behavior from the sub-Saharan applications based on data mentioned below to be able to reproduce the Dutch sexual network. Adjustments include the oldest age category, tendencies for more recent or concurrent partners by adjusting the promiscuity factors and steady relationship probabilities, and the age preference matrix, to correspond with the available data on Dutch sexual behavior, as described below.

People become available for a sexual relationship at the ‘age of sexual debut’, which is randomly drawn from a uniform distribution. The average age of sexual debut is 17 years for women and men, ranging from 12-22 years [[7-10](#_ENREF_7)]. When an individual is available, he or she can be selected by someone from the opposite sex who is at the end of his or her availability period. The duration of this period is drawn from an exponential distribution (Table S1). If the individual has not been chosen, he or she will select a partner from the pool of available persons of the opposite sex at the end of his or her availability period (uniformly distributed). Each time a relationship is formed or ended, a duration until the person becomes available for a new relationship is drawn from a predefined exponential distribution. For more information on the mechanisms and formulas, see also Hontelez *et al.* (2013)[[3](#_ENREF_3)]. The age- and sex-specific promiscuity factors (Table S1), which reflect the tendency of individuals to form relationships, were fine-tuned individually so that the model accurately reproduces the observed number of recent partners of men (Figure 1B).

Two types of sexual relationships are considered: long-term (‘steady’) relationships such as marriage (average duration of 40 years), and short-term (‘casual’) relationships (average duration of 1 year), both exponentially distributed. The type of relationship depends on the age of the male partner, and is defined as the (age-specific) probability of a steady relationship (Table S1). Every relationship starts with a sexual contact. After each contact, the time until a new sexual contact within the relationship is drawn from an exponential distribution. The mean frequency of sexual contacts depends on the age of the male partner. The sexual contact frequency within a relationship is on average once every 2 days for individuals up to 20 years old, decreasing to once per week for individuals from 21 to 35 years and once every two weeks for people older than 35 years ([[10](#_ENREF_10)], personal communication). The average duration of a relationship is drawn from an exponential distribution, depending on relationship type.

The probabilities of selecting a partner in a certain age class are defined in an age preference matrix (Table S2). When there is no partner available in that specific age class, the remaining age groups with a probability larger than 0 are used instead of resampling. The probabilities in the matrix were adjusted from sub-Saharan applications of STDSIM in order to reproduce the sexual age mixing patterns based on the national survey of the Rutgers WPF as reported by Schmid *et al*. (Figure 1C, 1D)[[11](#_ENREF_11)]. According to the data, the number of relationships between young people and much older individuals is negligible, hence we do not allow them to occur in the model at all. Per individual age group, we increased the probability of a relationship with partners with a similar age.

Independent from their sexual relationships, we modeled a high-risk group with more frequent one-off contacts (on average 2.25 contacts per month) to match the proportion of participants (11%) that reported to have 21 or more lifetime sexual partners in a Dutch survey [[8](#_ENREF_8),[10](#_ENREF_10)]. We used the STDSIM mechanisms originally designed for commercial sex work to this end.

By allowing for variation between individuals in the age of sexual debut and the number, type and overlap of sexual contacts as described above, heterogeneity in the population was taken into account. The fit of the sexual network was checked verified using the age differences in relationships [[11](#_ENREF_11)], the number of recent partners [[8](#_ENREF_8),[10](#_ENREF_10)] and the chlamydia prevalence ([[12](#_ENREF_12)], personal communication; see Figure 1).

**S1D: Chlamydia**

The transmission probability of chlamydia is assumed to be 0.45 per sexual contact, based on the model of Gray *et al.* [[13](#_ENREF_13)], which has contact frequencies similar to the Dutch survey data. The assumed average duration of infections is 52 weeks in women [[2](#_ENREF_2),[14-16](#_ENREF_14)] and 28 weeks in men [[14](#_ENREF_14),[17](#_ENREF_17)]. The susceptibility to re-infection is reduced by 30% after each successive infection, based on observations on ocular chlamydia [[18](#_ENREF_18)]. Chlamydia symptoms occur within 4 weeks (uniformly distributed) in 50% of the men and 30% of the women [[14](#_ENREF_14),[19](#_ENREF_19)]. Symptomatic men and women get tested with a test specificity of 99% and sensitivity of 98% [[20](#_ENREF_20)]. Patients receive treatment after a positive test result, leading to 100% cure of the infection, after which they become susceptible again. Their susceptibility is reduced by 30% after each successive infection, similar to individuals who naturally clear their infection. In our model, condoms have a protective effect against the transmission of chlamydia (yet do not protect against HPV, see main text) and were used in 7% of the sexual contacts in long-term relationships and 50% in short-term relationships and one-off contacts with a failure rate of 11.8%, based on the Dutch national sexual health survey [[8](#_ENREF_8)]. Data of the chlamydia prevalence came from the Chlamydia Screening Implementation (CSI) study in three regions in the Netherlands (Amsterdam, Rotterdam, and South Limburg) for 16 to 29-year old residents from 2008-2010 ([[12](#_ENREF_12)], personal communication). We used the prevalence estimates for the Limburg area as national level estimates, as was also done by Schmid *et al.* (2013)[[19](#_ENREF_19)].

**S1E: Alternative acquired immunity mechanism**

In our study, we also explored the possibility of an alternative mechanism for acquiring immunity after clearing an HPV infection, instead of a certain period of full immunity with Weibull distributed duration. In this alternative mechanism, we assumed that the susceptibility to re-infection decreases cumulatively after clearing each new infection. This way, the number of past infections directly influences the degree of protection. In this mechanism, we only estimated the Weibull shape of the duration of infection, the transmission probability per sexual contact, and the proportion of reduced susceptibility. To determine the best fitting model, we varied immunity from no immunity to 100% reduced susceptibility after each infection, and the transmission probability from 0% to 100% per sexual contact.

When assuming this mechanism, our results show again that a model with acquired immunity fits the HPV prevalence data for HPV-16 (χ*^2^* = 42.74, *p* < .001) and HPV-18 (χ*^2^* = 10.64, *p* = .014) much better than a model without acquired immunity, which underestimates the prevalence in women aged 25-33 years and overestimates the prevalence in women older than 39 years (Figure S1, Table S5). Again, a model with a Weibull distribution for the duration of infection instead of an exponential distribution fits better for HPV-16 (χ*^2^* = 47.04, *p* <.001), which shows large overestimations of the prevalence in women aged 18-28 years, and underestimations in women older than 29 years (Figure S1A). The corresponding Weibull shape for the duration is 0.50, equal to the base case mechanism. In this model, the susceptibility for re-infection is reduced by 58% after each infection (Table S6). The corresponding transmission probability is 5.3% (95% CI = 4.6; 6.1) per sexual contact. While this is the best fitting model, it still shows an overestimation in women aged 18-24 years and underestimation in women aged 25-33 years and older than 54 years (χ*^2^* = 19.14, *p* = .004).

For HPV-18, a model with a Weibull shape of 0.50 yields the best model fit to the observed data based on visual assessment and Pearson’s chi squared value (Figure S1B, Table S5), though the difference with a model with an exponentially distributed infection duration is not significant (χ*^2^* = 7.06, *p* = .07). In this model, the susceptibility for re-infection is reduced by 80% after each infection (Table S6). The corresponding transmission probability is 4.8% (95% CI= 4.2; 5.6) per sexual contact. The model shows no significant deviations from the data (χ*^2^* = 5.99, *p*=.42).

These results suggest that women need to clear about two subsequent HPV-16 infections in order to arrive at a similar level of acquired immunity (83%) after clearing one HPV-18 infection. Our best fitting model implies that around 45% will have an HPV-16 infection in their lifetime, and 20% an HPV-18 infection, similar to the base case immunity mechanism (Figure S2). Half of the women will never acquire an HPV-16 or -18 infection.

**S1F: Parameter values and goodness-of-fit of the best fitting models for HPV-16 and HPV-18**

We performed a two-dimensional grid search to determine combinations of transmission probabilities and mean duration of acquired immunity (or susceptibility reduction for the alternative mechanism) for each HPV-type, and the 5 pre-set shape parameters of the Weibull distributions of the durations of both infection and acquired immunity. In the models without immunity, we only fitted the transmission probability and Weibull shape for the duration of infection. The goodness-of-fit of the models was based on the log-likelihood, which we used in a likelihood ratio test to determine the best fitting model compared to the observed data (Table S4, [[21-23](#_ENREF_21)]). Table S5 shows the optimal parameter combinations and corresponding log-likelihoods when assuming no acquired immunity after clearing an infection; exponentially distributed durations; and Weibull distributed durations. For the best fitting models, we calculated Pearson’s chi-squared values to determine the model fit compared to the observed prevalence data (Table S5). The corresponding duration of infection and immunity are shown below in Tables S3 [[24](#_ENREF_24),[25](#_ENREF_25)] and S6, respectively.

**References**

1. Van der Ploeg CPB, Van Vliet C, De Vlas SJ, Ndinya-Achola JO, Fransen L, et al. (1998) STDSIM: A miscrosimulation model for decision support in STD control. Interfaces 28: 84-100.

2. Orroth KK, Freeman EE, Bakker R, Buve A, Glynn JR, et al. (2007) Understanding the differences between contrasting HIV epidemics in east and west Africa: results from a simulation model of the Four Cities Study. Sex Transm Infect 83 Suppl 1: i5-16.

3. Hontelez JA, Lurie MN, Barnighausen T, Bakker R, Baltussen R, et al. (2013) Elimination of HIV in South Africa through expanded access to antiretroviral therapy: a model comparison study. PLoS Med 10: e1001534.

4. Korenromp EL, Van Vliet C, Grosskurth H, Gavyole A, Van der Ploeg CP, et al. (2000) Model-based evaluation of single-round mass treatment of sexually transmitted diseases for HIV control in a rural African population. AIDS 14: 573-593.

5. Centraal Bureau voor de Statistiek. Available at: <http://www.cbs.nl>. Accessed March 2012.

6. United Nations, Department of Economic and Social Affairs, Population Division (2011). World Population Prospects: The 2010 Revision, CD-ROM Edition. .

7. De Graaf H, Meijer S, Poelman J, I V (2006) Sex below the age of 25: Sexual health of youth in the Netherlands in 2005. (in Dutch). Delft: The Netherlands: Rutgers Nisso Group.

8. Bakker F, De Graaf H, De Haas S, Kedde H, Kruijer H, et al. (2009) Sexual health in the Netherlands 2009 (in Dutch). Delft: the Netherlands.

9. SOA AIDS Nederland RW (2012) Belangrijkste conclusies Seks onder je 25e 2012.

10. Bakker F, Vanwesenbeeck, I. (2006) Sexual health in the Netherlands in 2006 [in Dutch]. Delft, the Netherlands.: Rutgers Nisso Group. Rutgers Nisso Groep studies, no.9. Rutgers Nisso Groep studies, no.9.

11. Schmid BV, Kretzschmar M (2012) Determinants of sexual network structure and their impact on cumulative network measures. PLoS Comput Biol 8: e1002470.

12. van den Broek IV, van Bergen JE, Brouwers EE, Fennema JS, Gotz HM, et al. (2012) Effectiveness of yearly, register based screening for chlamydia in the Netherlands: controlled trial with randomised stepped wedge implementation. BMJ 345: e4316.

13. Gray RT, Beagley KW, Timms P, Wilson DP (2009) Modeling the impact of potential vaccines on epidemics of sexually transmitted Chlamydia trachomatis infection. J Infect Dis 199: 1680-1688.

14. Kretzschmar M, Welte R, van den Hoek A, Postma MJ (2001) Comparative model-based analysis of screening programs for Chlamydia trachomatis infections. Am J Epidemiol 153: 90-101.

15. de Vries R, van Bergen JE, de Jong-van den Berg LT, Postma MJ, Group P-CS (2006) Systematic screening for Chlamydia trachomatis: estimating cost-effectiveness using dynamic modeling and Dutch data. Value Health 9: 1-11.

16. Gift TL, Gaydos CA, Kent CK, Marrazzo JM, Rietmeijer CA, et al. (2008) The program cost and cost-effectiveness of screening men for Chlamydia to prevent pelvic inflammatory disease in women. Sex Transm Dis 35: S66-75.

17. Low N, McCarthy A, Macleod J, Salisbury C, Campbell R, et al. (2007) Epidemiological, social, diagnostic and economic evaluation of population screening for genital chlamydial infection. Health Technol Assess 11: iii-iv, ix-xii, 1-165.

18. Bailey R, Duong T, Carpenter R, Whittle H, Mabey D (1999) The duration of human ocular Chlamydia Trachomatis infection is age dependent. Epidemiol Infect 123: 479-486.

19. Schmid BV, Over EA, van den Broek IV, Op de Coul EL, van Bergen JE, et al. (2013) Effects of population based screening for Chlamydia infections in the Netherlands limited by declining participation rates. PLoS One 8: e58674.

20. Coul ELM, Weenen TC, Van der Sande MAB, F. VdBIV (2009) Process evaluation of the Chlamydia Screening Implementation in the Netherlands: phase 1. Challenges and opportunities during preparation and first operational phase. RIVM report: 210261006/2009. Bilthoven, the Netherlands: RIVM.

21. Lenselink CH, Melchers WJ, Quint WG, Hoebers AM, Hendriks JC, et al. (2008) Sexual behaviour and HPV infections in 18 to 29 year old women in the pre-vaccine era in the Netherlands. PLoS One 3: e3743.

22. Bulkmans NW, Rozendaal L, Snijders PJ, Voorhorst FJ, Boeke AJ, et al. (2004) POBASCAM, a population-based randomized controlled trial for implementation of high-risk HPV testing in cervical screening: design, methods and baseline data of 44,102 women. International Journal of Cancer 110: 94-101.

23. Coupe VM, Berkhof J, Bulkmans NW, Snijders PJ, Meijer CJ (2008) Age-dependent prevalence of 14 high-risk HPV types in the Netherlands: implications for prophylactic vaccination and screening. British Journal of Cancer 98: 646-651.

24. Trottier H, Mahmud S, Prado JC, Sobrinho JS, Costa MC, et al. (2008) Type-specific duration of human papillomavirus infection: implications for human papillomavirus screening and vaccination. Journal of Infectious Diseases 197: 1436-1447.

25. Goodman MT, Shvetsov YB, McDuffie K, Wilkens LR, Zhu X, et al. (2008) Prevalence, acquisition, and clearance of cervical human papillomavirus infection among women with normal cytology: Hawaii Human Papillomavirus Cohort Study. Cancer Research 68: 8813-8824.

**Figure S1. Comparison of the observed and estimated age-specific HPV prevalence.** The estimated prevalence is given by the best fittings model when assuming no immunity (exponential distribution for the duration of infection) or different scenarios when assuming cumulatively decreasing susceptibility to re-infection after each infection (58% for HPV-16 and 80% for HPV-18). These scenarios include an exponentially distributed duration of infection and a Weibull distributed duration of infection (Weibull shape 0.50). ***(A)*** shows the results for HPV-16; ***(B)*** for HPV-18.

**Figure S2. Predicted cumulative number of HPV-16 *(A)*, HPV-18 *(B)*, and HPV-16/-18 infections in women.** The proportion of women with no lifetime infections slightly increased in women aged 65+ compared to women aged 50-64 years. This results from a cohort effect due to a combination of historical data on fertility rates and timing of an increase in migration (1965), and will only have a minimal effect on our estimates.

**Table S1. Sexual behavior parameters adjusted from previous STDSIM applications in order to reproduce to Dutch sexual network.**

**Table S2.** **Age preference matrix for men and women, adjusted to reproduce the observed age differences in relationships.**

**Table S3.** **The duration of infection when assuming different values for the shape parameter of the Weibull distribution of HPV-16 and HPV-18 infections.** The distribution is a weighted average based on the studies of Trottier *et al.*[24] and Goodman *et al.* [25]. Bold numbers indicate the Weibull shape and corresponding duration of infection for the best fitting models.

**Table S4. Observed high-risk HPV (hrHPV) prevalence with the type-specific fractions and the corresponding type-specific prevalence per age group.** The observed high-risk HPV prevalence is based on the studies of Lenselink *et al.* [21] and Bulkmans *et al.* [22]. By applying the fractions observed in Coupé *et al.* [23], we obtained the type-specific HPV-16 and HPV-18 prevalence.

**Table S5. Parameter values and goodness-of-fit for the best fitting HPV-16 and HPV-18 models of the different scenarios and both acquired immunity mechanisms.** The scenarios include no acquired immunity; exponentially distributed durations (Weibull shape = 1); and Weibull distributed durations.

**Table S6. Parameter values and the distribution of immunity durations for the best fitting HPV-16 and HPV-18 models under the base case and alternative immunity mechanism.** Scenarios include no acquired immunity; exponentially distributed duration (Weibull shape = 1); and Weibull distributed duration.
