# Supplementary figures and images for: The Role of Acquired Immunity in the Spread of Human Papillomavirus (HPV): Explorations with a Microsimulation Model
Source: PLoS One. 2015 Feb 2;10(2):e0116618. doi: 10.1371/journal.pone.0116618 (PMC4314063; doi:10.1371/journal.pone.0116618)

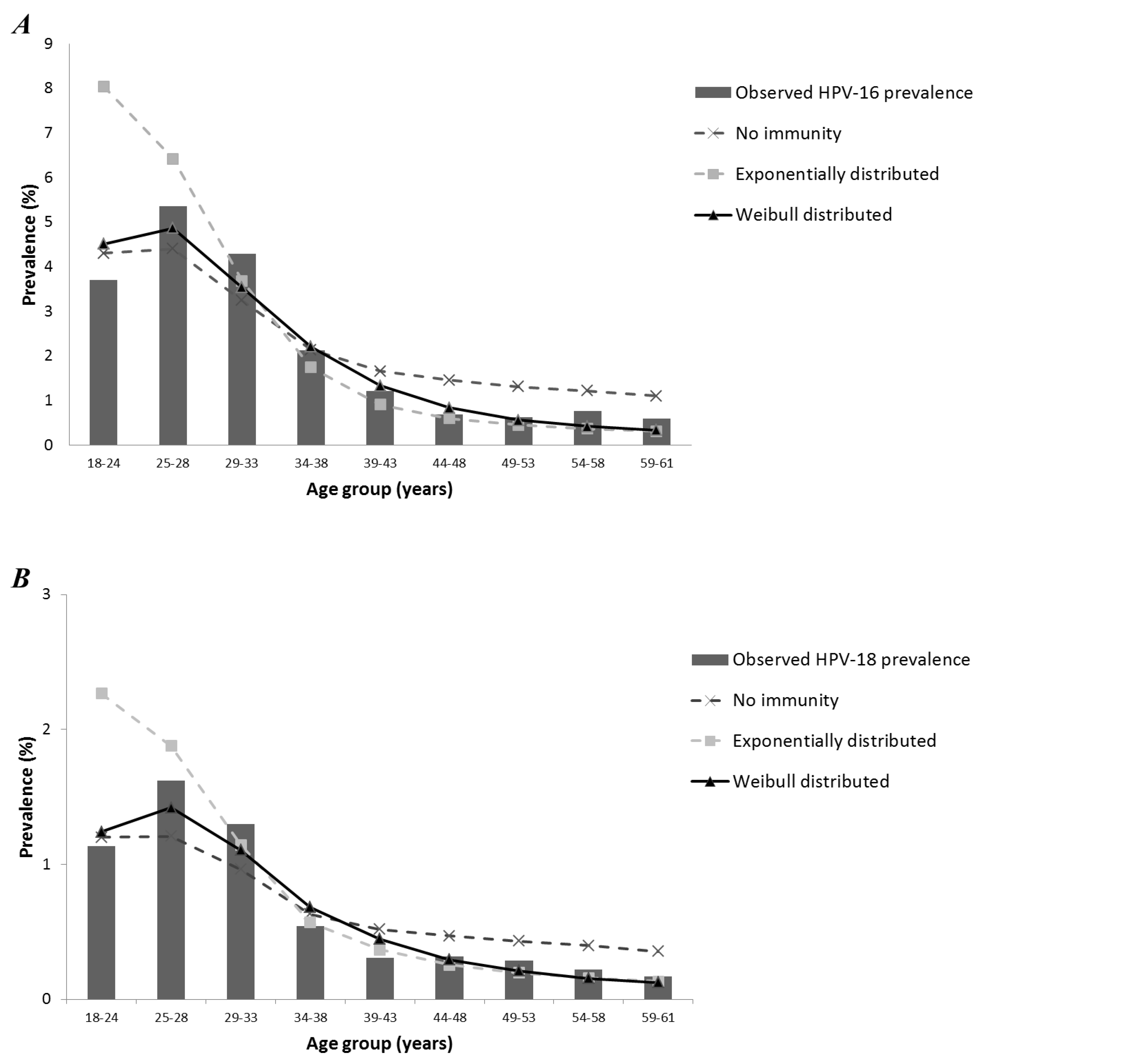

Supplement: S1 Fig — The estimated prevalence is given by the best fittings model when assuming no immunity (exponential distribution for the duration of infection) or different scenarios when assuming cumulatively decreasing susceptibility to re-infection after each infection (58% for HPV-16 and 80% for HPV-18). These scenarios include an exponentially distributed duration of infection and a Weibull distributed duration of infection (Weibull shape 0.50). (A) shows the results for HPV-16; (B) for HPV-18. (TIF) [file pone.0116618.s002.tif]

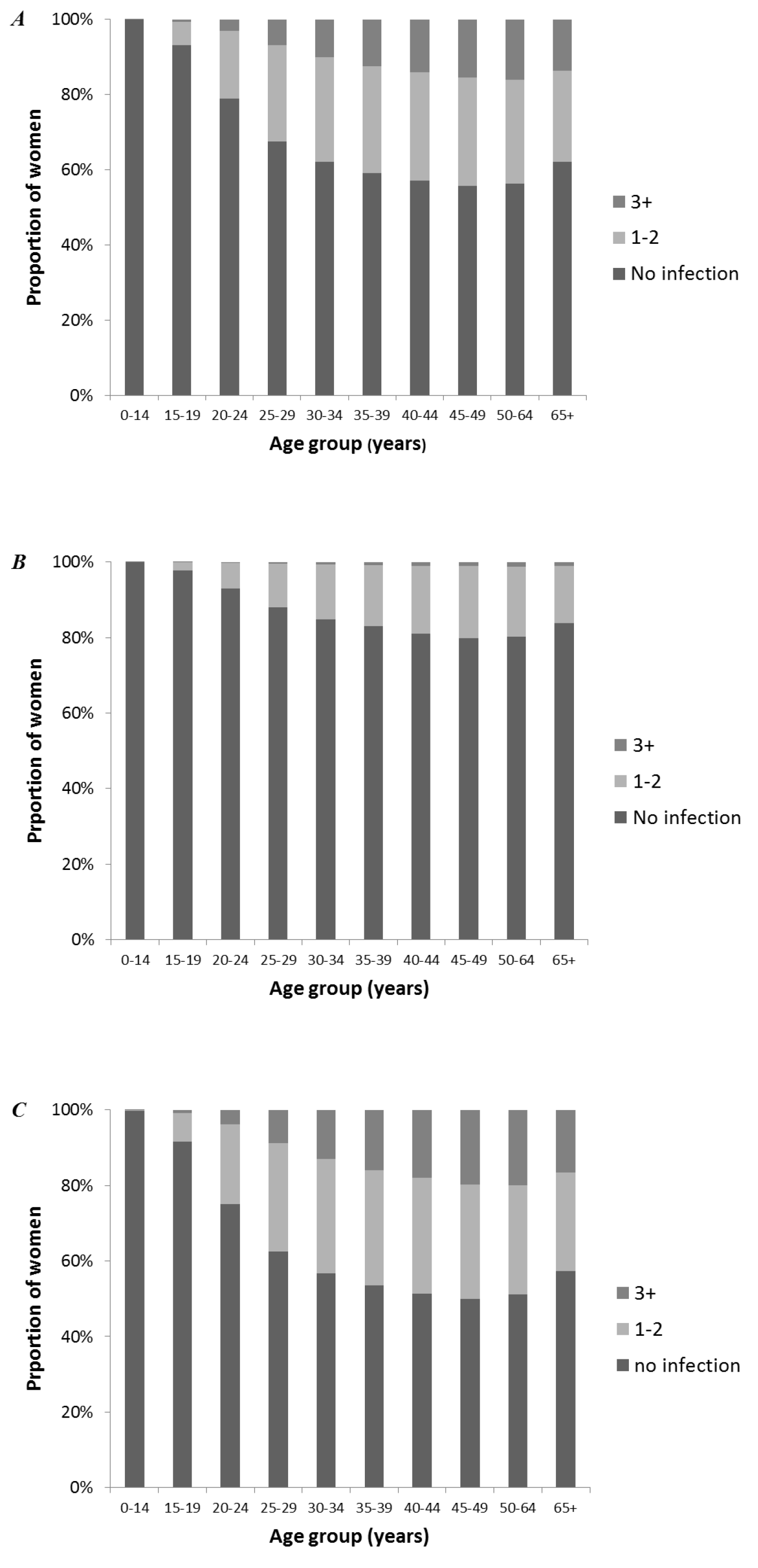

Supplement: S2 Fig — The proportion of women with no lifetime infections slightly increased in women aged 65+ compared to women aged 50–64 years. This results from a cohort effect due to a combination of historical data on fertility rates and timing of an increase in migration (1965), and will only have a minimal effect on our estimates. (TIF) [file pone.0116618.s003.tif]
