# Supplementary material for: The Role of Acquired Immunity in the Spread of Human Papillomavirus (HPV): Explorations with a Microsimulation Model
Source: PLoS One. 2015 Feb 2;10(2):e0116618. doi: 10.1371/journal.pone.0116618 (PMC4314063; doi:10.1371/journal.pone.0116618)
Supplement: S1 Table — (DOCX) [file pone.0116618.s004.docx]

**Table S1. Sexual behavior parameters adjusted from previous STDSIM applications in order to reproduce to Dutch sexual network.**

|  |  | **Men** | **Women** |
| --- | --- | --- | --- |
| Age-specific promiscuity | 0-14 | 2.9 | 2.6 |
|  | 15-19 | 2.9 | 3.8 |
|  | 20-24 | 7.2 | 5.5 |
|  | 25-29 | 6.1 | 2.6 |
|  | 30-34 | 5.6 | 2.9 |
|  | 35-39 | 2.3 | 0.7 |
|  | 40-44 | 1.8 | 0.5 |
|  | 45-49 | 1.6 | 0.3 |
|  | 50-64 | 1.6 | 0.3 |
|  | 65+ | 1.6 | 0.3 |
|  |  |  |  |
| Probability of a steady relationship | 0-14 | 0 | N/A* |
|  | 15-19 | 0.05 | N/A* |
|  | 20-24 | 0.3 | N/A* |
|  | 25-29 | 0.4 | N/A* |
|  | 30-34 | 0.5 | N/A* |
|  | 35-39 | 0.8 | N/A* |
|  | 40-44 | 0.9 | N/A* |
|  | 45-49 | 0.95 | N/A* |
|  | 50-64 | 0.95 | N/A* |
|  | 65+ | 0.95 | N/A* |
|  |  |  |  |
| Average time to availability (exponentially distributed) | single | 1 year | 1 year |
|  | casual relationship | 11 years | 20 years |
|  | steady relationship | 100 years | 100 years |
|  |  |  |  |
| Maximum duration availability period (uniformly distributed) |  | 1 year | 2.25 years |
|  |  |  |  |
| * determined by the age of the male partner | | | |
