# Supplementary material for: The Role of Acquired Immunity in the Spread of Human Papillomavirus (HPV): Explorations with a Microsimulation Model
Source: PLoS One. 2015 Feb 2;10(2):e0116618. doi: 10.1371/journal.pone.0116618 (PMC4314063; doi:10.1371/journal.pone.0116618)
Supplement: S2 Table — (DOCX) [file pone.0116618.s005.docx]

**Table S2.** **Age preference matrix for men and women, adjusted to reproduce the observed age differences in relationships.**

|  |  | Age of partner (years) | | | | | | | | | |
| --- | --- | --- | --- | --- | --- | --- | --- | --- | --- | --- | --- |
|  |  | 0-14 | 15-19 | 20-24 | 25-29 | 30-34 | 35-39 | 40-44 | 45-49 | 50-64 | 65+ |
| Age men (years) | 0-14 | 0.80 | 0.20 | 0 | 0 | 0 | 0 | 0 | 0 | 0 | 0 |
|  | 15-19 | 0.25 | 0.75 | 0 | 0 | 0 | 0 | 0 | 0 | 0 | 0 |
|  | 20-24 | 0 | 0.17 | 0.80 | 0.03 | 0 | 0 | 0 | 0 | 0 | 0 |
|  | 25-29 | 0 | 0 | 0.25 | 0.65 | 0.10 | 0 | 0 | 0 | 0 | 0 |
|  | 30-34 | 0 | 0 | 0.05 | 0.35 | 0.55 | 0.05 | 0 | 0 | 0 | 0 |
|  | 35-39 | 0 | 0 | 0 | 0.08 | 0.37 | 0.55 | 0 | 0 | 0 | 0 |
|  | 40-44 | 0 | 0 | 0 | 0 | 0.10 | 0.45 | 0.45 | 0 | 0 | 0 |
|  | 45-49 | 0 | 0 | 0 | 0 | 0 | 0.10 | 0.45 | 0.45 | 0 | 0 |
|  | 50-64 | 0 | 0 | 0 | 0 | 0 | 0 | 0.10 | 0.40 | 0.50 | 0 |
|  | 65+ | 0 | 0 | 0 | 0 | 0 | 0 | 0 | 0.05 | 0.20 | 0.75 |
|  |  |  |  |  |  |  |  |  |  |  |  |
| Age women (years) | 0-14 | 0.85 | 0.15 | 0 | 0 | 0 | 0 | 0 | 0 | 0 | 0 |
|  | 15-19 | 0.10 | 0.85 | 0.05 | 0 | 0 | 0 | 0 | 0 | 0 | 0 |
|  | 20-24 | 0 | 0.05 | 0.55 | 0.25 | 0.10 | 0.05 | 0 | 0 | 0 | 0 |
|  | 25-29 | 0 | 0 | 0 | 0.50 | 0.35 | 0.15 | 0 | 0 | 0 | 0 |
|  | 30-34 | 0 | 0 | 0 | 0 | 0.50 | 0.35 | 0.15 | 0 | 0 | 0 |
|  | 35-39 | 0 | 0 | 0 | 0 | 0 | 0.45 | 0.30 | 0.25 | 0 | 0 |
|  | 40-44 | 0 | 0 | 0 | 0 | 0 | 0 | 0.45 | 0.35 | 0.20 | 0 |
|  | 45-49 | 0 | 0 | 0 | 0 | 0 | 0 | 0 | 0.50 | 0.45 | 0.05 |
|  | 50-64 | 0 | 0 | 0 | 0 | 0 | 0 | 0 | 0 | 0.65 | 0.35 |
|  | 65+ | 0 | 0 | 0 | 0 | 0 | 0 | 0 | 0 | 0.25 | 0.75 |
