# Supplementary material for: The Role of Acquired Immunity in the Spread of Human Papillomavirus (HPV): Explorations with a Microsimulation Model
Source: PLoS One. 2015 Feb 2;10(2):e0116618. doi: 10.1371/journal.pone.0116618 (PMC4314063; doi:10.1371/journal.pone.0116618)
Supplement: S3 Table — The distribution is a weighted average based on the studies of Trottier et al. and Goodman et al. Bold numbers indicate the Weibull shape and corresponding duration of infection for the best fitting models. (DOCX) [file pone.0116618.s006.docx]

**Table S3.** **The duration of infection when assuming different values for the shape parameter of the Weibull distribution of HPV-16 and HPV-18 infections.** The distribution is a weighted average based on the studies of Trottier *et al.*[24] and Goodman *et al.* [25]. Bold numbers indicate the Weibull shape and corresponding duration of infection for the best fitting models.

|  | Weibull shape | Men | | | | | Women | | | |
| --- | --- | --- | --- | --- | --- | --- | --- | --- | --- | --- |
|  |  | Mean duration (months) | < 1 yr | | 1-4 yr | >4 yr | Mean duration (months) | < 1 yr | 1-4 yr | >4 yr |
| HPV-16 | 0.25 | 1268.4 | 49.9% | | 12.5% | 37.7% | 140.5 | 69.8% | 11.8% | 18.4% |
|  | **0.50** | **50.8** | **49.7%** | | **25.0%** | **25.3%** | **15.6** | **71.1%** | **20.6%** | **8.4%** |
|  | 1 | 17.6 | 49.4% | | 44.0% | 6.5% | 12.2 | 62.7% | 35.4% | 1.9% |
|  | 2 | 13.0 | 48.8% | | 51.2% | 0.0% | 11.7 | 56.2% | 43.8% | 0.0% |
|  | 4 | 12.1 | 47.7% | | 52.3% | 0.0% | 11.6 | 53.7% | 46.3% | 0.0% |
|  |  |  | | | | |  | | | |
| HPV-18 | 0.25 | 655.0 | | 55.7% | 12.7% | 31.6% | 201.8 | 66.5% | 12.2% | 21.3% |
|  | **0.50** | **26.2** | | **61.6%** | **23.7%** | **14.8%** | **17.2** | **69.3%** | **21.3%** | **9.4%** |
|  | 1 | 9.1 | | 73.3% | 26.2% | 0.5% | 12.2 | 62.7% | 35.3% | 1.9% |
|  | 2 | 6.7 | | 91.9% | 8.1% | 0.0% | 11.5 | 57.7% | 42.3% | 0.0% |
|  | 4 | 6.3 | | 100.0% | 0.0% | 0.0% | 11.3 | 57.2% | 42.8% | 0.0% |
