# Supplementary material for: The Role of Acquired Immunity in the Spread of Human Papillomavirus (HPV): Explorations with a Microsimulation Model
Source: PLoS One. 2015 Feb 2;10(2):e0116618. doi: 10.1371/journal.pone.0116618 (PMC4314063; doi:10.1371/journal.pone.0116618)
Supplement: S4 Table — The observed high-risk HPV prevalence is based on the studies of Lenselink et al. and Bulkmans et al. By applying the fractions observed in Coupé et al., we obtained the type-specific HPV-16 and HPV-18 prevalence. (DOCX) [file pone.0116618.s007.docx]

**Table S4. Observed high-risk HPV (hrHPV) prevalence with the type-specific fractions and the corresponding type-specific prevalence per age group.** The observed high-risk HPV prevalence is based on the studies of Lenselink *et al.* [21] and Bulkmans *et al.* [22]. By applying the fractions observed in Coupé *et al.* [23], we obtained the type-specific HPV-16 and HPV-18 prevalence.

| Age-group (years) | Observed hrHPV prevalence (%) | Type-specific fractions | | Type-specific HPV prevalence (%) | |
| --- | --- | --- | --- | --- | --- |
|  |  | HPV-16 | HPV-18 | HPV-16 | HPV-18 |
| 18-24 | 10.50 | 0.35 | 0.11 | 3.71 | 1.13 |
| 25-28 | 14.83 | 0.36 | 0.11 | 5.35 | 1.62 |
| 29-33 | 11.87 | 0.36 | 0.11 | 4.28 | 1.30 |
| 34-38 | 6.82 | 0.31 | 0.08 | 2.13 | 0.54 |
| 39-43 | 3.87 | 0.31 | 0.08 | 1.21 | 0.31 |
| 44-48 | 2.76 | 0.25 | 0.12 | 0.69 | 0.32 |
| 49-53 | 2.49 | 0.25 | 0.12 | 0.62 | 0.29 |
| 54-58 | 2.86 | 0.27 | 0.08 | 0.76 | 0.22 |
| 59-61 | 2.22 | 0.27 | 0.08 | 0.59 | 0.17 |
