# Supplementary material for: The Role of Acquired Immunity in the Spread of Human Papillomavirus (HPV): Explorations with a Microsimulation Model
Source: PLoS One. 2015 Feb 2;10(2):e0116618. doi: 10.1371/journal.pone.0116618 (PMC4314063; doi:10.1371/journal.pone.0116618)
Supplement: S5 Table — The scenarios include no acquired immunity; exponentially distributed durations (Weibull shape = 1); and Weibull distributed durations. (DOCX) [file pone.0116618.s008.docx]

**Table S5. Parameter values and goodness-of-fit for the best fitting HPV-16 and HPV-18 models of the different scenarios and both acquired immunity mechanisms.** The scenarios include no acquired immunity; exponentially distributed durations (Weibull shape = 1); and Weibull distributed durations.

|  | Wb inf | Wb imm | Log-likelihood | -2*Log-likelihood ratio | | -2*Log-likelihood ratio | | χ^2^ | p-value |
| --- | --- | --- | --- | --- | --- | --- | --- | --- | --- |
|  |  |  |  | Compared to no immunity | p-value | Compared to exponential distribution | p-value |  |  |
| HPV-16 | 1 | No imm | -53.97 |  |  |  |  |  |  |
|  | 1 | 1 | -58.65 | 9.36 | 0.053 |  |  |  |  |
|  | 0.50 | 0.25 | -32.03 | 43.88 | <.001 | 53.24 | <.001 | 15.26 | 0.009 |
|  | 0.50 | AM | -32.60 | 42.74 | <.001 | 47.04 | <.001 | 19.14 | 0.004 |
|  | 1 | AM | -56.12 | 4.3 | 0.23 |  |  |  |  |
|  |  |  |  |  |  |  |  |  |  |
| HPV-18 | 1 | No imm | -27.41 |  |  |  |  |  |  |
|  | 1 | 1 | -27.68 | 0.54 | 0.97 |  |  |  |  |
|  | 0.50 | 0.50 | -21.70 | 11.43 | 0.022 | 11.97 | 0.018 | 4.80 | 0.44* |
|  | 0.50 | 1 | -21.59 | 11.63 | 0.02 | 12.18 | 0.016 | 4.77 | 0.44* |
|  | 0.50 | 2 | -21.69 | 11.45 | 0.022 | 11.99 | 0.017 | 5.08 | 0.41* |
|  | 0.50 | 4 | -22.50 | 9.82 | 0.044 | 10.37 | 0.035 | 6.09 | 0.30* |
|  |  |  |  |  |  |  |  |  |  |
|  | 0.50 | AM | -22.09 | 10.64 | 0.014 | 7.06 | 0.07 | 5.99 | 0.42* |
|  | 1 | AM | -25.62 | 3.58 | 0.31 |  |  | 11.18 | 0.08* |
|  | 4 | AM | -25.62 | 3.58 | 0.31 |  |  | 10.48 | 0.11* |

Wb inf = Weibull shape infection duration; Wb imm = Weibull shape immunity duration; No imm = no immunity; AM = alternative mechanism; * indicates that the model does not differ significantly from the data, based on the chi-squared test.

The log-likelihood ratio test has 4 degrees of freedom for the base case immunity mechanism, and 3 degrees of freedom for the alternative mechanism. The chi-squared test has 5 degrees of freedom for the base case mechanism, and 6 degrees of freedom for the alternative mechanism.
