# Supplementary material for: The Role of Acquired Immunity in the Spread of Human Papillomavirus (HPV): Explorations with a Microsimulation Model
Source: PLoS One. 2015 Feb 2;10(2):e0116618. doi: 10.1371/journal.pone.0116618 (PMC4314063; doi:10.1371/journal.pone.0116618)
Supplement: S6 Table — Scenarios include no acquired immunity; exponentially distributed duration (Weibull shape = 1); and Weibull distributed duration. (DOCX) [file pone.0116618.s009.docx]

**Table S6. Parameter values and the distribution of immunity durations for the best fitting HPV-16 and HPV-18 models under the base case and alternative immunity mechanism.** Scenarios include no acquired immunity; exponentially distributed duration (Weibull shape = 1); and Weibull distributed duration.

|  | Wb inf | Wb imm | p | i | <1 year immune | >30 year immune |
| --- | --- | --- | --- | --- | --- | --- |
| HPV-16 | 1 | No immunity | 3.1% | - | - | - |
|  | 1 | 1 | 19.4% | 3.96 years | 22.3% | 0.0% |
|  | **0.50** | **0.25** | **6.9%** | **111.65 years** | **49.4%** | **20.3%** |
|  | **0.50** | **AM** | **5.3%** | **58.40%** | - | - |
|  | 1 | AM | 6.8% | 25.30% | - | - |
|  |  |  |  |  |  |  |
| HPV-18 | 1 | No immunity | 3.4% | - | - | - |
|  | 1 | 1 | 13.4% | 11.46 years | 8.4% | 7.3% |
|  | **0.50** | **0.50** | **6.7%** | **115.18 years** | **12.3%** | **48.6%** |
|  | **0.50** | **1** | **7.7%** | **43.25 years** | **2.3%** | **50.0%** |
|  | **0.50** | **2** | **7.6%** | **28.67 years** | **0.1%** | **42.3%** |
|  | **0.50** | **4** | **9.0%** | **28.12 years** | **0.0%** | **41.7%** |
|  | **0.50** | **AM** | **4.8%** | **79.70%** | - | - |
|  | 1 | AM | 7.6% | 40.80% | - | - |
|  | 4 | AM | 8.6% | 30.80% | - | - |

Wb inf=Weibull shape infection duration; Wb imm=Weibull shape immunity duration; p=transmission probability per sexual contact;i=mean immunity duration; AM=alternative mechanism. Bold numbers indicate the best fitting models.

All women that clear an HPV infection will become immune. The percentages of women being immune for <1 year and >30 years are shown; the remaining women are immune for a period of 1-30 years.
